# Impact of Postpartum Hemorrhage in Postpartum Depressive and Anxiety Symptoms in Twin Delivery: A Retrospective Cohort Study

**DOI:** 10.3390/jcm15082953

**Published:** 2026-04-13

**Authors:** Hui Ye, Yilan Tian, Guolin He, Kaige Pei, Lin Li

**Affiliations:** 1 Department of Obstetrics and Gynecology, West China Second University Hospital, Sichuan University, Number 20, Third Section of People’s South Road, Chengdu 610000, China; huaxiyehui@foxmail.com (H.Y.); hxeytyl@foxmail.com (Y.T.); heguolin19@163.com (G.H.); peikaige0727@163.com (K.P.); 2 Key Laboratory of Birth Defects and Related Diseases of Women and Children, Sichuan University, Ministry of Education, Chengdu 610000, China

**Keywords:** twin, postpartum hemorrhage, depression, anxiety

## Abstract

**Objectives**: This study aimed to investigate the association between postpartum hemorrhage (PPH) and the risk of postpartum depressive and anxiety symptoms. **Methods**: A retrospective cohort study of twin delivery was conducted at a tertiary women’s and children’s hospital from May 2022 to May 2024. Participants were screened using the Edinburgh Postnatal Depression Scale and the Self-Rating Anxiety Scale. Participants with a pre-existing psychiatric history were excluded. The association between PPH and psychological outcomes was analyzed using multivariate logistic regression. **Results**: Among the 885 participants, 57 (6.44%) experienced PPH. The prevalence of postpartum depressive symptoms was significantly higher in the PPH group (29.8%) compared to the non-PPH group (17.75%, *p* = 0.02). After full adjustment for confounders, PPH remained significantly associated with an increased odds of postpartum depressive symptoms (OR = 2.45; 95% CI: 1.24–4.85; *p* = 0.01). Subgroup analyses indicated this association was particularly strong in women with a normal BMI and those who delivered via cesarean section. In contrast, no statistically significant association was found between PPH and postpartum anxiety symptoms in any of the adjusted models. **Conclusions**: In twin pregnancies, postpartum hemorrhage is an independent risk factor for postpartum depressive symptoms, but not for anxiety symptoms. These findings highlight the critical need for targeted and enhanced postpartum mental health screening and follow-up care for mothers of twins who experience PPH to facilitate early detection and intervention for depression.

## 1. Introduction

The perinatal period represents a critical phase of life transition, characterized by profound physiological, psychological, and social transformations in mothers [1]. Depression and anxiety disorders are reported to affect 19% and 13% of postpartum individuals [2,3], respectively, and represent leading causes of global disability, with substantial impacts on individual well-being and healthcare systems [4,5,6]. Maternal depression and anxiety are associated with diminished maternal self-care and impaired infant caregiving and bonding [7,8,9], and are further linked to delayed cognitive, emotional, and social development in children [10,11]. Numerous studies indicate that women experiencing postpartum depressive symptoms have an increased risk of recurrent depression later in life [12]. Concerningly, suicide has been recognized as a leading cause of maternal mortality in the postpartum period [13,14]. Therefore, understanding the determinants of postpartum depression and anxiety symptoms and implementing preventive interventions to mitigate their occurrence is of critical importance.

Postpartum hemorrhage (PPH) remains one of the leading causes of maternal morbidity and mortality worldwide, accounting for approximately 11% of all maternal deaths [15]. In addition, it is associated with a high incidence of psychological burden symptoms among both women and their partners [16]. A multicenter cohort study in Australia reported that 11% of women who experienced PPH developed postpartum depression at 2 months postpartum, rising to 13% at 4 months [17]; however, this study lacked a control group. The development of postpartum depression may be influenced by multiple factors, including antenatal depression and anxiety. Furthermore, Ricbourg and colleagues conducted a case–control study with a limited sample size (20 cases and 20 controls) to examine the psychological impact of PPH on women and found no significant association between PPH and postpartum depressive symptoms [16]. In contrast, Wang et al. demonstrated in a singleton pregnancy study that women with PPH were significantly more likely to screen positive for postpartum depressive symptoms compared to those without PPH (16.4% vs. 11.7%, *p* = 0.016) [18]. Twin gestations are associated with a higher risk of adverse maternal outcomes, including severe morbidity and mortality [19]. PPH occurs more frequently in twin pregnancies than in singleton gestations [20,21]. There is limited data regarding the association between PPH and postpartum psychological disorders, particularly in the case of twin deliveries. Hence, we carried out a retrospective cohort study to investigate the impact of PPH on postpartum depressive and anxiety symptoms among parturients with twin pregnancies.

## 2. Materials and Methods

### 2.1. Study Design and Study Population

We conducted a hospital-based retrospective review of twin deliveries at West China Second University Hospital, Sichuan University—a tertiary women’s and children’s hospital—between 1 May 2022, and 1 May 2024. Depression and anxiety screening was performed within 4 weeks postpartum. Postpartum anxiety symptoms were assessed using the Self-Rating Anxiety Scale (SAS), and depressive symptoms were evaluated using the Edinburgh Postnatal Depression Scale (EPDS). Inclusion criteria were as follows: (1) age ≥ 18 years, (2) confirmed twin pregnancy, (3) gestational age at delivery ≥ 28 weeks, (4) liveborn infants without congenital malformations, (5) no personal or family history of psychiatric disorders, and (6) complete data on PPH and depression and anxiety screening. All puerperal women meeting these criteria were eligible for inclusion. The study protocol was approved by the Institutional Review Board of West China Second University Hospital, Sichuan University (YXKY2025061). The methodology followed the principles of the Declaration of Helsinki. Patient consent was waived due to the retrospective nature of the study. The main exposure was PPH. According to widely accepted clinical guidelines, PPH is defined as blood loss of at least 500 mL following vaginal delivery and at least 1000 mL following cesarean delivery. For vaginal delivery, blood loss was estimated by the obstetrician and midwife using visual assessment and calibrated drapes. For cesarean delivery, the surgeon and anesthesiologist jointly estimated intraoperative blood loss using suction canisters and weighed sponges and drapes.

### 2.2. Screening for Postpartum Depressive Symptoms

The primary outcome was postpartum depressive symptoms, assessed using EPDS. The EPDS comprises ten items, each rated on a 4-point scale (0–3), yielding a total score ranging from 0 to 30. Higher scores indicate greater severity of depressive symptoms. The reliability and validity of the Chinese version of the EPDS as a screening tool for postpartum depression have been well established [22]. In this study, a score greater than or equal to nine was considered an indicator of postpartum depressive symptoms [23].

### 2.3. Screening for Postpartum Anxiety Symptoms

The second outcome was postpartum anxiety symptoms, assessed using the SAS. The SAS consists of 20 items, each designed to reflect a common anxiety-related symptom. Respondents are instructed to select the response that best corresponds to their experiences over the past week from four response options—“Never or rarely,” “Some of the time,” “Most of the time,” and “Almost all of the time,”—which are scored on a 4-point scale ranging from 1 to 4, with 5 items reverse-scored. The total raw score is calculated by summing the item scores and then multiplied by 1.25 to yield the standard score. A standard score of 50 or below is considered within the normal range, whereas scores above 50 indicate the presence of clinically significant anxiety symptoms [24]. All women were asked to complete both the EPDS and SAS questionnaires during the same visit; no participant was exempted from completing either scale based on her responses to the other.

### 2.4. Assessment of Covariates

Baseline data were extracted from the participants’ medical records, including sociodemographic data such as maternal age, parity, pregnancy weight and height at delivery, and hospitalization cost. Pregnancy body mass index (BMI) was calculated by dividing pregnancy weight (kg) by the square of height (m^2^). Information on primiparity, delivery mode, gestational age, blood loss volume, chorionicity type and the use of assisted reproductive technology and infant congenital malformations was also obtained from medical records.

### 2.5. Statistical Analysis

Data are presented as means ± standard deviations for continuous variables and as frequencies (percentages) for categorical variables. Independent samples t-tests were used to compare continuous variables between groups, while χ^2^ tests were employed to assess differences in proportions. Multivariate logistic regression models were applied to estimate the association between postpartum hemorrhage (PPH) and the risk of postpartum depression or anxiety. To evaluate potential effect modification, two approaches were adopted—(a) stratified analyses by maternal age, pregnancy BMI, gestational week at delivery, and cesarean section, and (b) sequential evaluation of potential effect modifiers in separate multivariate logistic regression models, including maternal age, BMI, gestational age at delivery, parity, mode of delivery, hospitalization cost, and postpartum anxiety or depression status—with each model adjusted for a pre-specified set of different confounding factors. Statistical significance was determined at a *p*-value threshold of <0.05. All analyses were conducted using SPSS software (version 29.0.2.0 (20)).

## 3. Results

### 3.1. Baseline Characteristics

**Figure 1:**
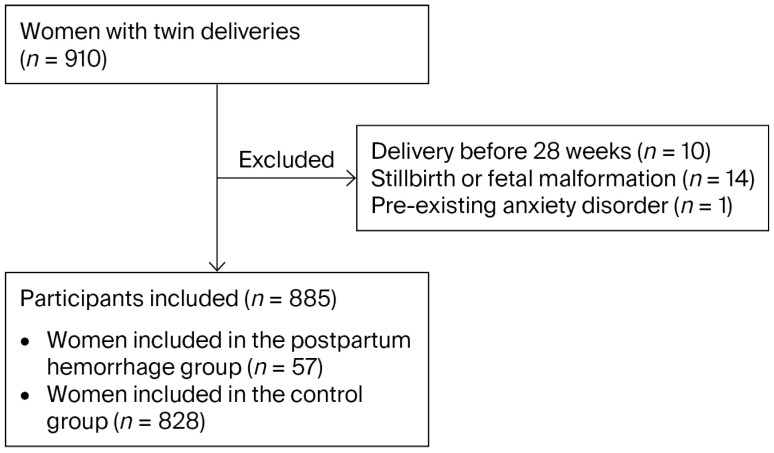
Flowchart of the screening process.

During the study period, a total of 910 pregnant women with twin pregnancies completed assessments using standardized depression and anxiety rating scales. Of these, 10 participants were excluded due to gestational week at delivery less than 28 weeks, one was excluded due to a pre-existing diagnosis of anxiety disorder, and an additional 14 were excluded owing to stillbirth or fetal malformations at delivery. The final analytical sample comprised 885 puerperal women, among whom 164 (18.5%) screened positive for postpartum depressive symptoms and 160 (18.1%) for postpartum anxiety symptoms. The participant selection process is illustrated in Figure 1.

Based on postpartum blood loss volume, 57 women (6.44%) were diagnosed with PPH according to standard clinical criteria and were assigned to the PPH group; the remaining 828 women (93.56%) without PPH formed the control group. Compared with the control group, the PPH group was significantly older (*p* = 0.01) and incurred higher hospitalization costs (*p* < 0.01). The prevalence of postpartum depressive symptoms was 29.8% in the PPH group versus 17.75% in the control group. Regarding the timing of postpartum screening, the median time from delivery to assessment was 14 days (interquartile range: 7–21 days). No statistically significant difference was observed between the postpartum hemorrhage group and the control group (*p* = 0.94). Women experiencing PPH exhibited a significantly higher prevalence of postpartum depressive symptoms than those without PPH (*p* = 0.02), as shown in Table 1.

### 3.2. PPH and Depressive Symptoms

**Figure 2:**
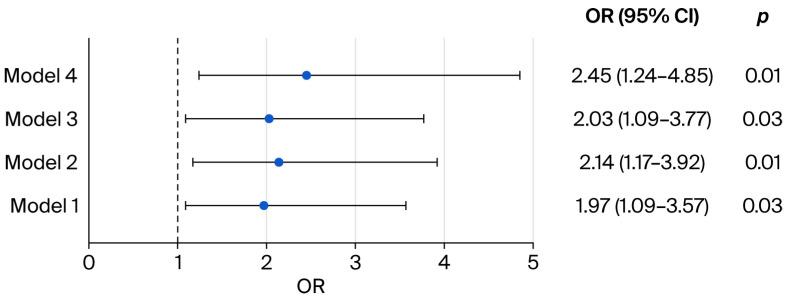
Association between postpartum hemorrhage and the risk of postpartum depression in women with twin delivery. Model 1: unadjusted. Model 2: adjusted for age, BMI and gestational week at delivery. Model 3: adjusted for age, BMI, gestational week at delivery, parity, mode of delivery, and hospitalization cost. Model 4: adjusted for age, BMI, gestational week at delivery, parity, mode of delivery, hospitalization cost and postpartum anxiety.

Four logistic regression analyses evaluating the relationship between PPH and postpartum depressive symptoms are shown in Figure 2. In the initial unadjusted model (Model 1), PPH was significantly linked to a greater likelihood of developing postpartum depressive symptoms (OR = 1.97; 95% CI: 1.09–3.57; *p* = 0.03). After accounting for maternal age, BMI and gestational week at delivery in Model 2, the estimated risk increased slightly (OR = 2.14; 95% CI: 1.17–3.92; *p* = 0.01). Model 3 incorporated additional adjustments for parity, mode of delivery, and hospitalization cost, resulting in a significant association (OR = 2.03; 95% CI: 1.09–3.77; *p* = 0.03). Model 4 further included postpartum anxiety, and the effect of PPH remained statistically significant (OR = 2.45; 95% CI: 1.24–4.85; *p* = 0.01), suggesting that PPH independently contributes to an elevated risk of postpartum depressive symptoms even after adjusting for a range of potential confounders.

Given that maternal age, BMI, gestational week at delivery and mode of delivery are known risk factors for postpartum depressive symptoms, we assessed whether these variables modified the association between PPH and postpartum depressive symptoms. Stratified analyses revealed a statistically significant association between postpartum hemorrhage (PPH) and postpartum depressive symptoms among women who delivered by cesarean section (OR = 2.01; 95% CI: 1.10–3.64; *p* = 0.02) (Table 2). The subgroup analyses were prespecified but remain exploratory in nature, and their findings should therefore be interpreted with caution.

### 3.3. PPH and Anxiety Symptoms

Similar to the analysis of depressive symptoms, we employed a four-step modeling approach to systematically examine the association between PPH and postpartum anxiety symptoms. The results showed that after progressive adjustment for potential confounders, no statistically significant association was observed between PPH and postpartum anxiety symptoms in any of the models (*p* ≥ 0.05), with detailed estimates presented in Table A1. Furthermore, subgroup analyses stratified by maternal age, gestational week at delivery, and mode of delivery consistently yielded non-significant associations (shown in Table A2). Collectively, these findings indicate that, following comprehensive adjustment for a range of potential confounding factors, there is no statistically significant association between postpartum hemorrhage and postpartum anxiety symptoms.

## 4. Discussion

To the best of our knowledge, this is the first large-scale study to evaluate the association between postpartum hemorrhage and both depression and anxiety in parturients with twin pregnancies. Multiple analytical models consistently demonstrated a significant association between postpartum hemorrhage and increased odds of postpartum depressive symptoms. However, no statistically significant association was found between postpartum hemorrhage and postpartum anxiety in this population.

A cohort study involving 273 women found that the prevalence rates of elevated anxiety symptoms ranged from 13.8% to 20.0%, while depressive symptoms were reported by 14.5% to 16.4% of participants [25]. The incidence of postpartum anxiety symptoms in our study was similar to previous research, while postpartum depression incidence was higher. This may be related to the study period partially overlapping with the COVID-19 pandemic and the inclusion of women who delivered twins.

Regarding the design and results, the present study bears similarities to a singleton delivery study carried out by Wang and his colleagues. Adopting a retrospective cohort study design, the aforesaid study also revealed that the positive rate of postpartum depression screening among women who had experienced postpartum hemorrhage was significantly higher than that among those who had not (16.4% vs. 11.7%, *p* = 0.016) [18]. The incidence of postpartum depression among women with twin pregnancies is higher than that in those with singleton pregnancies, potentially attributable to the elevated risk of postpartum hemorrhage observed in twin pregnancies. However, a nested cohort study (n = 446), based on two population-based cohorts in Uppsala, Sweden, found no significant association between PPH and postpartum depression symptoms [26]. Meanwhile, a meta-analysis of nine studies (n = 934,432) demonstrated that women who experienced PPH were at significantly increased risk of developing postpartum depression compared to those without PPH (OR = 1.28, 95% CI: 1.13–1.44, *p* < 0.001), with substantial heterogeneity across studies (I^2^ = 98.9%) [27]. Their research results suggest that the association between PPH and postpartum depression is further amplified among women with a prior history of depression or anxiety. Different from their study, in the design of this study, patients with a history of depression and anxiety were excluded, yet this trend was still observed.

The findings of this study may be interpreted through several interrelated mechanisms. First, synthetic oxytocin is routinely administered during twin deliveries to prevent postpartum hemorrhage; however, accumulating evidence suggests that its use is associated with an increased risk of postpartum depressive disorders [28,29]. Second, twin pregnancies are characterized by a higher incidence and severity of postpartum hemorrhage, which predisposes women to anemia—a condition linked to multiple emotional disturbances such as fatigue and mood lability, both of which are recognized precursors to depressive symptoms [26,30]. Third, women experiencing PPH are more likely to report adverse childbirth experiences and exhibit elevated rates of post-traumatic stress symptoms, factors that may significantly contribute to the development of postpartum depression [31]. Finally, PPH often results in prolonged hospitalization and increased medical expenditures, thereby imposing substantial psychological stress and exacerbating emotional vulnerability during the postpartum period [18,32].

The strength of our study lies in the inclusion of a large sample of mothers who delivered twins, which enhances the statistical power and improves the generalizability of our findings. Additionally, by excluding women with a prior history of anxiety, depression, or other psychiatric disorders, we reduced potential confounding effects and minimized selection bias. Nevertheless, several limitations warrant consideration. First, the use of screening instruments—namely, the EPDS and the SAS—does not equate to a clinical diagnosis; these tools are designed to detect symptom severity and should be followed by structured psychiatric assessments for definitive diagnosis. Second, the mode of delivery may influence postpartum psychological outcomes. Notably, our analysis did not account for the fact that many women with twin pregnancies at our institution are of advanced maternal age and conceived via assisted reproductive technology, both of which are independently associated with a higher likelihood of elective cesarean delivery—a potential pathway influencing mental health outcomes. Third, because prenatal depressive and anxiety symptoms were missing for a substantial proportion of participants, these variables could not be incorporated into the multivariable models. To mitigate residual confounding, we prospectively excluded individuals with any personal or family history of psychiatric disorders. Lastly, unmeasured or unknown confounders, including psychosocial stressors, perinatal complications, newborn sex, low birth weight, and postpartum sleep deprivation, may have affected the interpretation of our results.

## 5. Conclusions

In summary, this study found that PPH was associated with increased odds of postpartum depressive symptoms among parturients with twin pregnancies. The results of this research may facilitate health visitors in clarifying the focal points of postpartum follow-up for twin-bearing parturients experiencing PPH. To prevent the emergence of postpartum depressive symptoms, the influence of PPH on such symptoms should be meticulously considered, particularly in the case of parturients with twin pregnancies.

## Figures and Tables

**Table 1:** Baseline characteristics and pregnancy-related data of women with twin delivery.

	PPH Group	Control Group	*p*-Value
*n* (%)	57 (6.44)	828 (93.56)	-
Age, mean (SD), y	33.53 (4.24)	32.02 (3.89)	0.01
Blood loss, median (IQR), mL	1200 (1000–1450)	400 (400–500)	<0.01
BMI, median (IQR), kg/m^2^	26.48 (25.11–28.12)	27.26 (25.12–28.84)	0.65
BMI < 24, *n* (%)	5 (8.77)	113 (13.65)	0.30
Hospitalization cost, median (IQR), RMB	22,072.03 (17,856.71–28,966.31)	16,165.89 (13,983.30–19,267.68)	<0.01
Primiparity, *n* (%)	45 (78.95)	692 (83.57)	0.37
Postpartum screening timing, median (IQR), day	14 (8–21)	14 (7–21)	0.94
Gestational week at delivery, mean (SD), week	36.14 (1.32)	35.78 (1.80)	0.05
Cesarean delivery, *n* (%)	56 (98.25)	812 (98.07)	0.93
Dichorionicity, *n* (%)	47 (82.46)	626 (75.60)	0.24
Assisted reproductive technology, *n* (%)	36 (63.16)	508 (61.35)	0.79
Depression symptoms, *n* (%)	17 (29.82)	147 (17.75)	0.02
Anxiety symptoms, *n* (%)	10 (17.54)	150 (18.12)	0.91

**Table 2:** Associations between PPH and postpartum depression stratified by maternal age, BMI, and mode of delivery.

Characteristics	PPH	Control	OR	*p*-Value
Age	57	828		
<35	11/38 (28.95)	112/627 (18.50)	2.01 (0.96–4.20)	0.06
≥35	6/19 (31.58)	35/201(17.41)	2.01 (0.70–5.76)	0.19
Gestational week at delivery				
<37	8/26 (30.77)	88/433 (20.32)	1.43 (0.50–4.04)	0.50
≥37	9/31 (29.03)	59/395 (14.94)	2.07 (0.87–4.91)	0.10
Cesarean section				
Yes	17/56 (30.36)	145/812 (17.86)	2.01 (1.10–3.64)	0.02
No	0/1 (0)	2/16 (12.50)	-	-

## Data Availability

The data presented in this study are available on request from the corresponding author.

## Abbreviations

The following abbreviations are used in this manuscript:

PPH	Postpartum hemorrhage
BMI	Body mass index
CI	Confidence interval
OR	Odd ratio
EPDS	Edinburgh Postnatal Depression Scale
SAS	Self-Rating Anxiety Scale

## Appendix A

**Table A1:** Association between postpartum hemorrhage and the risk of postpartum anxiety in women with twin delivery.

Characteristics	Control	PPH	*p*-Value
No. of participants	828	57	-
Model 1	Ref.	0.96 (0.48–1.95)	0.91
Model 2	Ref.	1.01 (0.49–2.05)	1.00
Model 3	Ref.	0.94 (0.45–1.94)	0.86
Model 4	Ref.	0.60 (0.27–1.36)	0.22

Model 1: unadjusted. Model 2: adjusted for age, BMI and gestational week at delivery. Model 3: adjusted for covariates in Model 2 and parity, mode of delivery, and hospitalization cost. Model 4: adjusted for covariates in Model 3 and postpartum depression.

**Table A2:** Associations between PPH and postpartum anxiety stratified by maternal age, BMI, and mode of delivery.

Characteristics	PPH	Control	OR	*p*-Value
Age	57	828		
<35	6/38 (15.79)	116/627 (18.50)	0.99 (0.92–1.06)	0.69
≥35	4/19 (21.05)	34/201 (16.92)	1.27 (0.39–4.14)	0.69
Gestational week at delivery				
<37	4/26 (15.38)	90/433 (20.79)	0.76 (0.22–2.67)	0.67
≥37	6/31 (19.35)	60/395 (15.19)	1.46 (0.57–3.75)	0.43
Cesarean section				
Yes	10/56 (17.86)	147/812 (18.10)	1.41 (0.55–3.61)	0.47
No	0/1 (0)	3/16 (18.75)	-	-
